# Isolation, Antimicrobial Resistance Phenotypes, and Virulence Genes of *Bordetella bronchiseptica* From Pigs in China, 2018–2020

**DOI:** 10.3389/fvets.2021.672716

**Published:** 2021-06-08

**Authors:** Yue Zhang, Hao Yang, Long Guo, Mengfei Zhao, Fei Wang, Wenbo Song, Lin Hua, Lei Wang, Wan Liang, Xibiao Tang, Zhong Peng, Bin Wu

**Affiliations:** ^1^State Key Laboratory of Agricultural Microbiology, College of Veterinary Medicine, Huazhong Agricultural University, Wuhan, China; ^2^MOST International Research Center for Animal Disease, Cooperative Innovation Center for Sustainable Pig Production, Huazhong Agricultural University, Wuhan, China; ^3^Diagnostic Center of Animal Diseases, Wuhan Keqian Biology Co., Ltd, Wuhan, China; ^4^MARA Key Laboratory of Prevention and Control Agents for Animal Bacteriosis, Institute of Animal Husbandry and Veterinary, Hubei Academy of Agricultural Sciences, Wuhan, China

**Keywords:** *Bordetella bronchiseptica*, isolation, antimicrobial resistance, virulence factors encoding genes, pigs

## Abstract

*Bordetella bronchiseptica* is a leading cause of respiratory diseases in pigs. However, epidemiological data of *B. bronchiseptica* in pigs particularly in China, the largest pig rearing country in the world is still limited. We isolated 181 *B. bronchiseptica* strains from 4259 lung samples of dead pigs with respiratory diseases in 14 provinces in China from 2018 to 2020. The average isolation rate of this 3-year period was 4.25% (181/4259). Antimicrobial susceptibility testing performed by disc diffusion method revealed that most of the *B. bronchiseptica* isolates in this study were resistant to ampicillin (83.98%), while a proportion of isolates were resistant to cefotaxime (30.39%%), chloramphenicol (12.71%), gentamicin (11.60%), florfenicol (11.60%), tetracycline (8.84%), amoxicillin (8.29%), tobramycin (6.63%), ceftriaxone (4.97%), and cefepime (0.55%). There were no isolates with resistant phenotypes to imipenem, meropenem, polymyxin B, ciprofloxacin, enrofloxacin, and amikacin. In addition, ~13.18% of the isolates showed phenotypes of multidrug resistance. Detection of antimicrobial resistance genes (ARGs) by PCR showed that 16.57% of the *B. bronchiseptica* isolates in this study was positive to *aac(3)-IV*, while 3.87%, 2.21%, 1.10%, 0.55%, 0.55%, and 0.55% of the isolates were positive to *aac6'-Ib, rmtA, bla*_TEM_, *bla*_SHV_, *oqxB*, and *tetA*, respectively. Detection of virulence factors encoding genes (VFGs) by conventional PCR showed that over 90% of the pig *B. bronchiseptica* isolates in this study were positive to the five VFGs examined (*fhaB*, 97.24%; *prn*, 91.16%; *cyaA*, 98.34%; *dnt*, 98.34%; *betA*, 92.82%). These results demonstrate *B. bronchiseptica* as an important pathogen associated with pig respiratory disorders in China. The present work contributes to the current understanding of the prevalence, antimicrobial resistance and virulence genes of *B. bronchiseptica* in pigs.

## Introduction

*Bordetella bronchiseptica* is an aerobic, motile, gram-negative rod, or coccobacillus belonging to genus *Bordetella*. It is an important pathogenic bacterium in agriculture and in veterinary medicine (1). In veterinary medicine, *B. bronchiseptica* is a leading cause of many respiratory infections including rhinitis, tracheitis, bronchitis, and pneumonia in a wide spectrum of animals (2). It can also enhance respiratory colonization of *Streptococcus suis* and *Haemophilus parasuis*, promote disease caused by *S. suis*, and interact with porcine reproductive and respiratory syndrome virus (PRRSV) and swine influenza virus (SIV) to increase severity of respiratory disease (3). While rarely to be reported, *B. bronchiseptica* is also potentially involved in infections in humans, and human cases are frequently associated with direct contact with infected animals such as swine, dog, rabbit and/or cat (4–6). Similar to the other members belonging to genus *Bordetella*, many *B. bronchiseptica* produces several important virulence factors, including filamentous hemagglutinin, and protein toxins, adenylate cyclase toxin, pertussis toxin, dermonecrotic toxin as well as type III secretion system (T3SS) and effector proteins, contributing to its pathogenesis (7, 8).

In swine, *B. bronchiseptica* is proposed as a main causative agent of porcine respiratory disease complex (PRDC) and atrophic rhinitis; both of which are economically-important diseases in pig industry (9, 10). Continuously monitoring the prevalence, antimicrobial resistance (AMR) and virulence profiles of *B. bronchiseptica* in pigs are beneficial for the prevention and control of swine bordetellosis. However, the relevant data are still limited. China is the largest pig-farming and pork consuming country in the world. Although the outbreak of African Swine Fever in August 2018 caused a huge loss of pigs in China, there are still more than 406 million pigs rearing in China in 2020 (11). To understand the current epidemiological and microbiological characteristics such as the antimicrobial resistance profiles of *B. bronchiseptica* isolates from pigs in China, we performed bacterial isolation of *B. bronchiseptica* strains from lung samples of dead pigs with a history of respiratory disorders in China from 2018 to 2020 in this study. These isolates were characterized by testing the antimicrobial susceptibility and detecting the antimicrobial resistance genes (ARGs) as well as virulence encoding genes (VFGs).

## Materials and Methods

### Study Design, Sample Collection, and Ethic Statement

**Figure 1:**
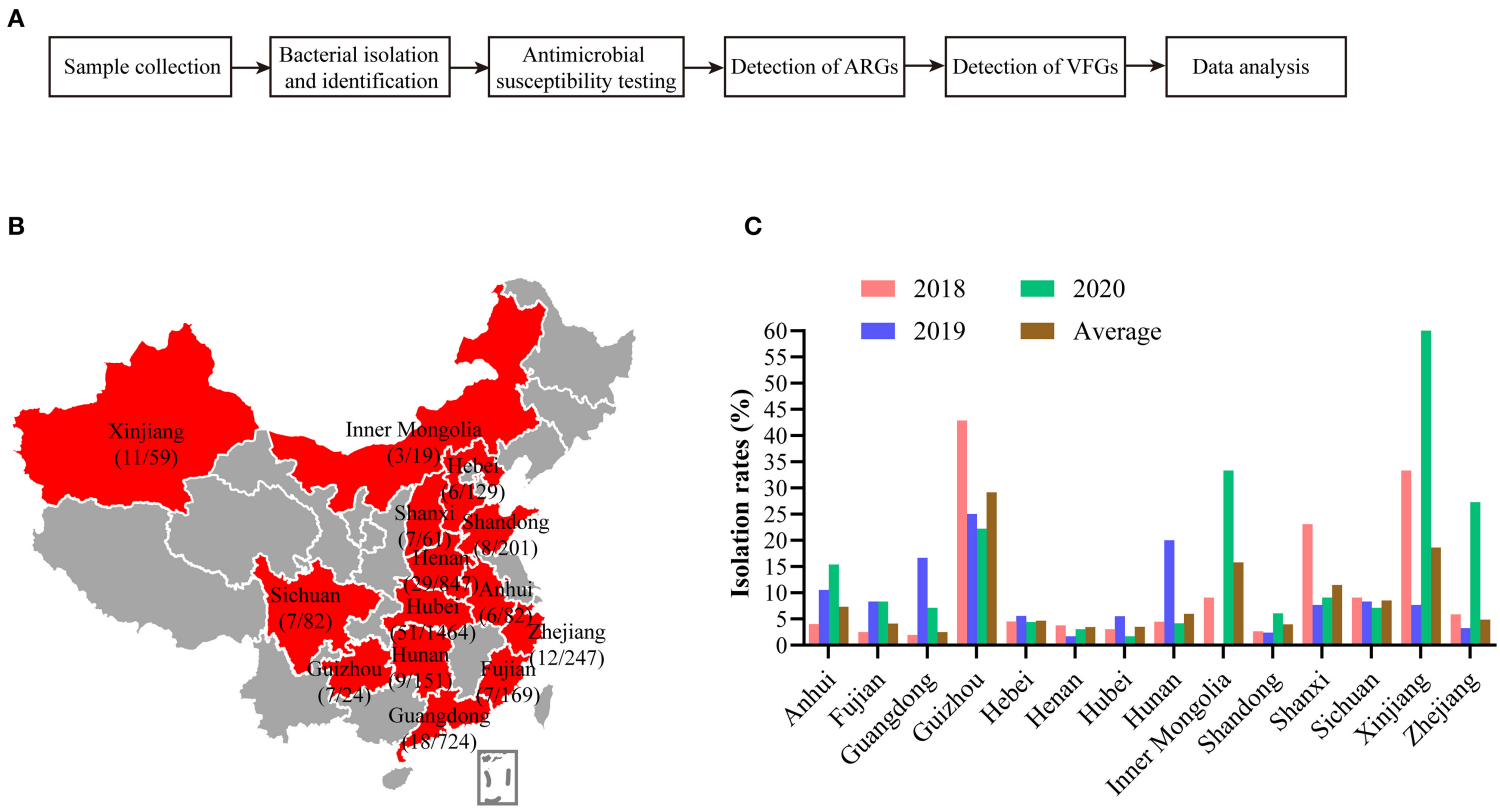
Study design and isolation of *B. bronchiseptica* from swine lung samples in different regions in China from 2018 to 2020. **(A)** Shows study design of this work; **(B)** shows the geographic sites of sample collection. Numbers of *B. bronchiseptica* from the samples as well as numbers of samples collected from each of the provinces are shown; **(C)** shows the isolation rates of *B. bronchiseptica* from swine lung samples in different regions in China from 2018 to 2020.

Study design was shown in Figure 1A. From 2018 to 2020, a total of 4259 lung samples (3022 samples in 2018, 841 samples in 2019, 396 samples in 2020) from 14 provinces (Guangdong, Henan, Hubei, Shandong, Fujian, Hebei, Zhejiang, Hunan, Anhui, Sichuan, Shanxi, Inner Mongolia, Xinjiang, Guizhou) in China were used for *B. bronchiseptica* isolation and identification (Figure 1B). All of the clinical samples used in this study were submitted by veterinarians/or the farm owners to the Veterinary Diagnostic Laboratory of Huazhong Agricultural University (Wuhan, China) for routine testing.

### Bacterial Isolation and Identification

Collected samples (~10 grams per sample) were cut into pieces and lysed in sterile 0.9% normal saline by using a TissueLyser II (QIAGEN, Venlo, Netherlands). Thereafter, tissue homogenates of each sample were streak-plated onto one tryptic soy agar (TSA; Becton, Dickinson and Company, MD, USA) containing 10 μg/ml nicotinamide adenine dinucleotide (NAD; Sigma, St. Louis, MO) and 10% new-born bovine serum. The agar plates were incubated at 37°C for 24~48 h. Isolates growing on the plates were then purified and cultured following the standard methods used for bacterial identification (12). On each of the agar plates, five colonies with similar morphological characteristics to *B. bronchiseptica* [small circular glistening or rough colonies with 0.5 to 1.0 mm in diameter after 48 h of incubation in air at 37°C (4)] were selected for biochemical test. Presumptive isolates of *B. bronchiseptica* were finally confirmed using polymerase chain reaction (PCR) assay amplifying the species-specific gene *fla* with the primers listed in Table 1 (26). Considering *B. bronchiseptica* possesses only one serotype (27), we therefore chose one colony confirmed by both PCR and biochemical tests (positive for *fla* and displaying similar biochemical characteristics to *B. bronchiseptica*) to represent *B. bronchiseptica* strain recovered for its corresponding sample.

**Table 1:** Primers used in the present study.

**Primers**	**Sequences (5^**′**^-3^**′**^)**	**Product size (bp)**	**Annealing temperature (^**°**^C)**	**Description**	**References**
Bacterial species identification genes					
Fla1	TGGCGCCTGCCCTATC	237	56	*B. bronchiseptica* identification	(13)
Fla2	AGGCTCCCAAGAGAGAAA				
Antimicrobial resistance genes					
SHV1	CCCTGTTAGCCACCCTGCCG	829	62	Detection of *bla*_SHV_	(14)
SHV2	CGTTGCCAGTGCTCGATCAGC				
CTXM1	GCTGTTGTTAGGAAGTGTGCCGC	798	61	Detection of *bla*_CTX−M_	(14)
CTXM2	GCCGCCGACGCTAATACATC				
TEM1	GTATTCAACATTTCCGTGTCG	854	56	Detection of *bla*_TEM_	(14)
TEM2	CCAATGCTTAATCAGTGAGGC				
VIM-1	GATGGTGTTTGGTCGCATA	390	57	Detection of *bla*_VIM_	(15)
VIM-2	CGAATGCGCAGCACCAG				
NDM-1	GGTTTGGCGATCTGGTTTTC	621	56	Detection of *bla*_NDM−1_	(15)
NDM-2	CGGAATGGCTCATCACGATC				
MOX-1	GCTGCTCAAGGAGCACAGGAT	520	59	Detection of *OMX*	(16)
MOX-2	CACATTGACATAGGTGTGGTGC				
AAC-1	GTTACACCGGACCTTGGA	674	55	Detection of *aac(3)-IV*	(17)
AAC-2	AACGGCATTGAGCGTCAG				
Aac6-1	TTGCGATGCTCTATGAGTGGCTA	482	58	Detection of *aac6'-Ib*	(18)
Aac6-2	CTCGAATGCCTGGCGTGTTT				
RmtA-1	ATGAGCTTTGACGATGCCCTA	756	53	Detection of *rmtA*	(19)
RmtA-2	TCACTTATTCCTTTTTATCATG				
QnrS1	CGACGTGCTAACTTGCGTGATA	537	58	Detection of *qnrS*	(20)
QnrS2	TACCCAGTGCTTCGAGAATCAG				
OqxA-1	GATCAGTCAGTGGGATAGTTT	670	52	Detection of *oqxA*	(21)
OqxA-2	TACTCGGCGTTAACTGATTA				
OqxB-1	TTCTCCCCCGGCGGGAAGTAC	512	61	Detection of *oqxB*	(22)
OqxB-2	CTCGGCCATTTTGGCGCGTA				
TetA-1	GTAATTCTGAGCACTGTCGC	937	56	Detection of *tetA*	(23)
TetA-2	CTGCCTGGACAACATTGCTT				
TetB-1	CTCAGTATTCCAAGCCTTTG	416	44	Detection of *tetB*	(23)
TetB-2	CTAAGCACTTGTCTCCTGTT				
FloR-1	CACGTTGAGCCTCTATAT	868	52	Detection of *floR*	(23)
FloR-2	ATGCAGAAGTAGAACGCG				
CatA11	CCACCGTTGATATATCCC	623	55	Detection of *catA1*	(17)
CatA12	CCTGCCACTCATCGCAGT				
CatA21	TTTGCCCTTTATCGTCAGC	486	55	Detection of *catA2*	This study
CatA22	GCGGTCACCTTCCTGCT				
Mcr-1	CGGTCAGTCCGTTTGTTC	309	58	Detection of *mcr-1*	(24)
Mcr-2	CTTGGTCGGTCTGTAGGG				
Virulence factors encoding genes					
FhaB-1	GCGCAGAACATCACCAATG	475	59	Filamentous haemagglutinin encoding gene *fhaB*	(25)
FhaB-2	TGAAATACTCCATGGCGGAC				
Prn-1	GACCTCGCTCAGTCGATC	555	59	Pertactin encoding gene *prn*	
Prn-2	GAAGACATTCATGCGGAACAG				
CyaA-1	CTACGAGCAGTTCGAGTTTC	377	59	Adenylate cyclase-haemolysin toxin encoding gene *cyaA*	
CyaA-2	TATTCATGTCGCCGTCGTA				
Dnt-1	TGATCCTGCAGTGGTTGATC	491	59	Dermonecrotic toxin encoding gene *dnt*	
Dnt-2	ATCGGCATACGCCAGATC				
BteA-1	TGTTGAGCAACAACGTCAATC	474	59	Bordetella type-III secretion system effector A encoding gene *bteA*	
BteA-2	TATGCAGGTCTTCGAGGTTC				

### Antimicrobial Susceptibility Testing

Antimicrobial susceptibility of the *B. bronchiseptica* isolates was tested by using Disk diffusion method following the Clinical and Laboratory Standards Institute (CLSI) antimicrobial susceptibility testing standards (28). Briefly, purified overnight-cultured colonies of *B. bronchiseptica* were picked up from TSA plates and resuspended in sterile 0.9% normal saline to 0.5 McFarland standard. The suspension was then prepared by swabbing on Mueller-Hinton (MH) agar (Sigma-Aldrich, 102 St. Louis, MO) using sterile swabs. After dry for ~5 min, disks containing specific antibiotics (Hangzhou Microbial Reagent, Hangzhou, China) were dispensed onto the plates. All plates were finally incubated overnight at an incubation temperature of 37°C. A total of 16 types of antibiotics including amikacin [AMK; 30 μg], gentamicin [GEN; 10 μg], tobramycin [TOB; 10 μg], ceftriaxone [CRO; 30 μg], cefotaxime [CTX; 30 μg], cefepime [CPM; 30 μg], imipenem [IPM; 10 μg], meropenem [MRP; 10 μg], enrofloxacin [ENR; 10 μg], ciprofloxacin [CIP; 5 μg], chloramphenicol [CHL; 30 μg], florfenicol [FLO; 30 μg], amoxicillin [AMX; 20 μg], ampicillin [AMP; 10 μg], tetracycline [TET; 30 μg], and polymyxin B [PMB; 300 IU] were tested. The zone diameter values were measured and the results were interpreted according to CLSI document (28). As clinic breakpoints specific to *B. bronchiseptica* are limited available (2), we thereby used breakpoints to *Enterobacteriaceae* published in CLSI document M100 for result-interpretation in this study. Breakpoints used are listed in Table 2. *Escherichia coli* ATCC®^*^ 25922 was used as quality control.

**Table 2:** Zone Diameter Breakpoints (mm) used in the present study.

**Antibiotics**		**Amikacin**	**Gentamicin**	**Tobramycin**	**Ceftriaxone**	**Cefotaxime**	**Cefepime**	**Imipenem**	**Meropenem**
Z.^*^	R	≤ 14	≤ 12	≤ 12	≤ 13	≤ 14	≤ 14	≤ 13	≤ 19
D.	I	15~22	13~14	13~14	14~20	15~22	15~17	14~15	20~22
B.	S	≥23	≥15	≥15	≥21	≥23	≥18	≥15	≥23
**Antibiotics**		**Enrofloxacin**	**Ciprofloxacin**	**Chloramphenicol**	**Florfenicol**	**Amoxicillin**	**Ampicillin**	**Tetracycline**	**Polymyxin B**
Z.	R	≤ 15	≤ 15	≤ 12	≤ 12	≤ 17	≤ 19	≤ 14	≤ 8
D.	I	16~20	16~20	13~17	13~17	18~20	20~22	15~18	8~11
B.	S	≥ 21	≥ 21	≥ 18	≥18	≥ 21	≥ 23	≥19	≥12

* *Zone Diameter Breakpoints (Z.D.B.) were defined as sensitive (S), intermediately resistant (I), or resistant (R) with reference to CLSI (CLSI document M100, 28^th^ Edition)*.

### Detection of Antimicrobial Resistance Genes

PCR assays were performed to detect the presence of putative genes conferring resistance to aminoglycosides [*aac(3)-IV, aac6'-Ib, rmtA*], β-lactams (*bla*_VIM_, *bla*_NDM−1_, *bla*_TEM_, *bla*_SHV_, *bla*_CTX−M_, *MOX*), quinolones (*qnrS, oqxA, oqxB*), phenicols (*floR, catA1, catB1*), tetracyclines (*tetA, tetB*), and polymyxins (*mcr-1*) in each of the *B. bronchiseptica* isolates with the primers listed in Table 1. PCR assays were performed in a 20-μL reaction mixture comprised of 2-μL bacterial DNA, each of the forward and reverse primers 1-μL, 2 × Taq Master Mix (Dye Plus) 10-μL, DMSO 2-μL, and ddH_2_O 4-μL. The cycling conditions were 94°C for 5 min, followed by 35 cycles consisting of denaturation for 30 s at 94°C, annealing for 30 s at 52~63°C, and extension for 30 s at 72°C, and a final extension at 72°C for 5 min. PCR products were analyzed by electrophoresis on a 1% agarose gel. Genomic DNAs extracted from our previously sequenced multidrug resistant *E. coli* strain RXD033 (GenBank accession no. SQQZ00000000) (29) and drug-sensitive bacterium *Pasteurella multocida* strain HND05 (GenBank accession no. PPWG00000000) (30) were used as positive and negative controls, respectively.

### Detection of Virulence Factors Encoding Genes

The presence of five well-characterized VFGs, including the filamentous haemagglutinin encoding gene *fhaB*, the pertactin encoding gene *prn*, the adenylate cyclase-haemolysin toxin encoding gene *cyaA*, the dermonecrotic toxin encoding gene *dnt*, and the Bordetella type-III secretion system effector A encoding gene *bteA* in each of the isolates were examined by PCR with primers listed in Table 1, as described previously (25). PCR assays were performed in a 20-μL reaction mixture comprised of 2-μL bacterial DNA, each of the forward and reverse primers 1-μL, 2 × Taq Master Mix (Dye Plus) 10-μL, DMSO 2-μL, and ddH_2_O 4-μL. The cycling conditions were 94°C for 5 min, followed by 35 cycles of 94°C for 30 s, 59°C for 30 s and 72°C for 30 s, and a final extension at 72°C for 5 min. Our laboratory stored *B. bronchiseptica* strain HH0809 (31) and the sterile ddH_2_O were included as the positive and negative controls, respectively. PCR products were analyzed by electrophoresis on a 1% agarose gel.

### Statistical Analysis

We used SAS version 9.0 (SAS Institute Inc.) software to perform statistical analyses in this study, as described previously (26). Univariate association between variables and isolation rates of *B. bronchiseptica* was determined by using univariate ordinary logistic regression analysis. *P* <0.05 was considered to be significant.

## Results

### *B. bronchiseptica* Isolation and Identification

From 2018 to 2020, we isolated a total of 181 *B. bronchiseptica* strains (4.25%) from 4259 lung samples of dead pigs with respiratory diseases. The isolation rates of *B. bronchiseptica* over the 3 years were 3.51, 5.47, and 7.32%, respectively. Rates of isolation across different provinces in China ranged from 2.49 to 29.17% (Figures 1B,C). Biochemical tests revealed that *B. bronchiseptica* isolates could not ferment fructose, glucose, mannitol, maltose, rhamnose, and lactose; the methyl red (MR), voges-proskauer (VP), and indole reactions were negative. It is positive testes for oxidase and catalase.

### Antimicrobial Susceptibility Testing

**Figure 2:**
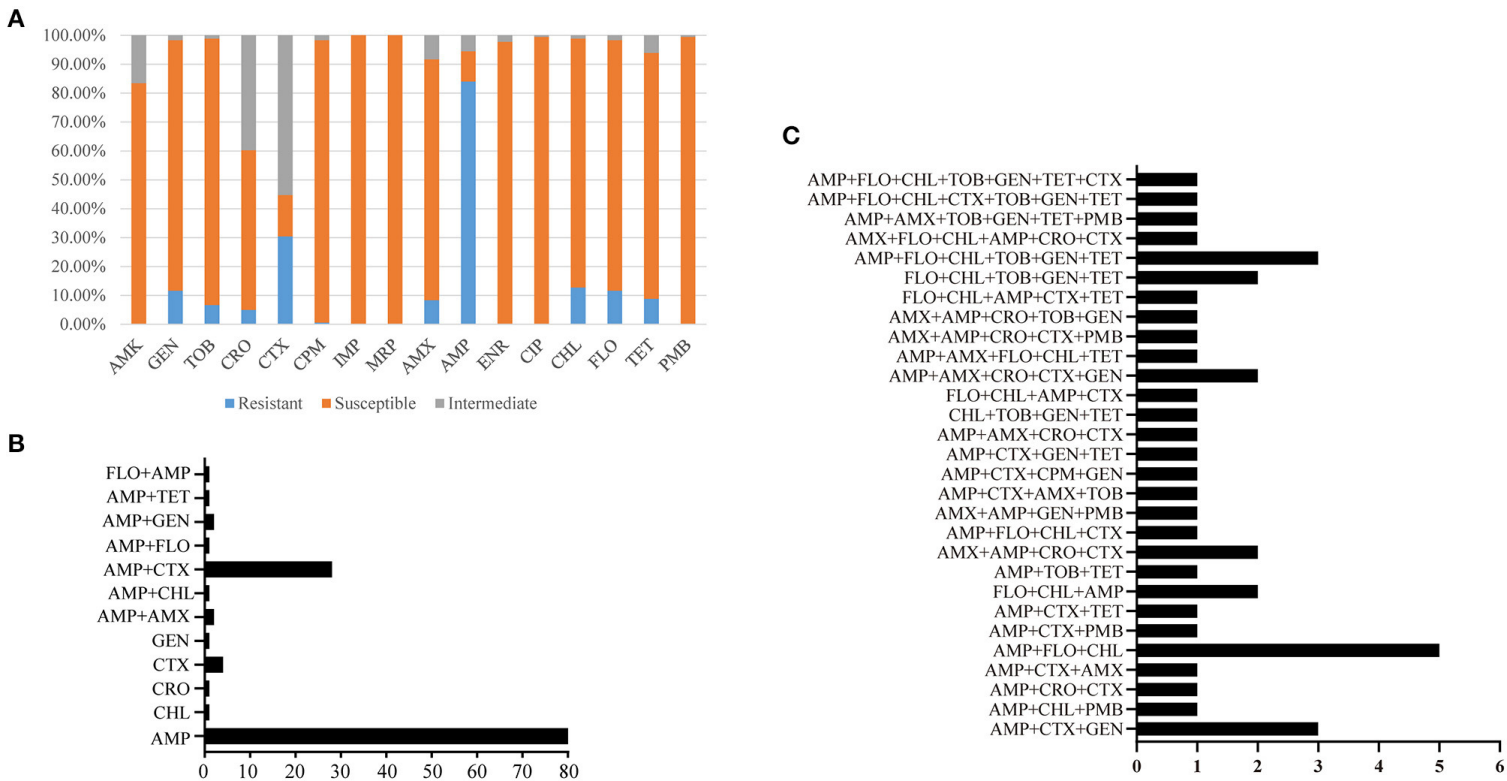
Resistance phenotypes of *B. bronchiseptica* from pigs in China. **(A)** Shows percent isolates susceptible or resistant to the 16 kinds of antibiotics tested; **(B,C)** display the number of isolates with different resistance patterns. In **(B,C)**, X axes show the number of *B. bronchiseptica* strains while Y axes indicate different resistance patterns. AMK, amikacin; GEN, gentamicin; TOB, tobramycin; CRO, ceftriaxone; CTX, cefotaxime; CPM, cefepime; IPM, imipenem; MRP, meropenem; ENR, enrofloxacin; CIP, ciprofloxacin; CHL, chloramphenicol; FLO, florfenicol; AMX, amoxicillin; AMP, ampicillin; TET, tetracycline; PMB, polymyxin B.

Antimicrobial susceptibility testing (AST) revealed that 9.39% (*n* = 17) of the *B. bronchiseptica* isolates recovered in this study were susceptible to all of the 16 types of the antibiotics tested while the remaining 90.61% (*n* = 164) of the isolates were resistant to at least one type of the antibiotics. All of the *B. bronchiseptica* isolates recovered in this study were susceptible to imipenem (100%, *n* = 181), meropenem (100%, *n* = 181), and polymyxin B (100%, *n* = 181); more than 80% of the *B. bronchiseptica* isolates were susceptible to ciprofloxacin (99.45%, *n* = 180), cefepime (97.79%, *n* = 177), enrofloxacin (97.79%, *n* = 177), tobramycin (92.27%, *n* = 167), gentamicin (86.74%, *n* = 157), florfenicol (86.74%, *n* = 157), chloramphenicol (86.19%, *n* = 156), tetracycline (85.08%, *n* = 154), amikacin (83.43%, *n* = 151), and amoxicillin (83.43%, *n* = 151) (Figure 2A). Approximately 55.25% (*n* = 100) of the *B. bronchiseptica* isolates were susceptible to ceftriaxone, while only 14.36% (*n* = 26) and 10.50% (*n* = 19) of the *B. bronchiseptica* isolates were susceptible to cefotaxime and ampicillin, respectively (Figure 2A). Among the 164-drug resistant *B. bronchiseptica* isolates, resistance rates to 1 type, 2 types, 3 types, 4 types, 5 types, 6 types, and 7 types of drugs were 53.05% (*n* = 87), 23.17% (*n* = 38), 7.32% (*n* = 12), 6.10% (*n* = 10), 4.88% (*n* = 8), 3.66% (*n* = 6), and 1.22% (*n* = 2), respectively (Figure 2B). Approximately 50.00% (*n* = 82), 26.83% (*n* = 44), 17.07% (*n* = 28), 9.76% (*n* = 16), and 4.88% (*n* = 8) of the isolates were resistant to at least 2 types, 3 types, 4 types, 5 types, and 6 types of the antibiotics tested, respectively (Figures 2B,C).

**Figure 3:**
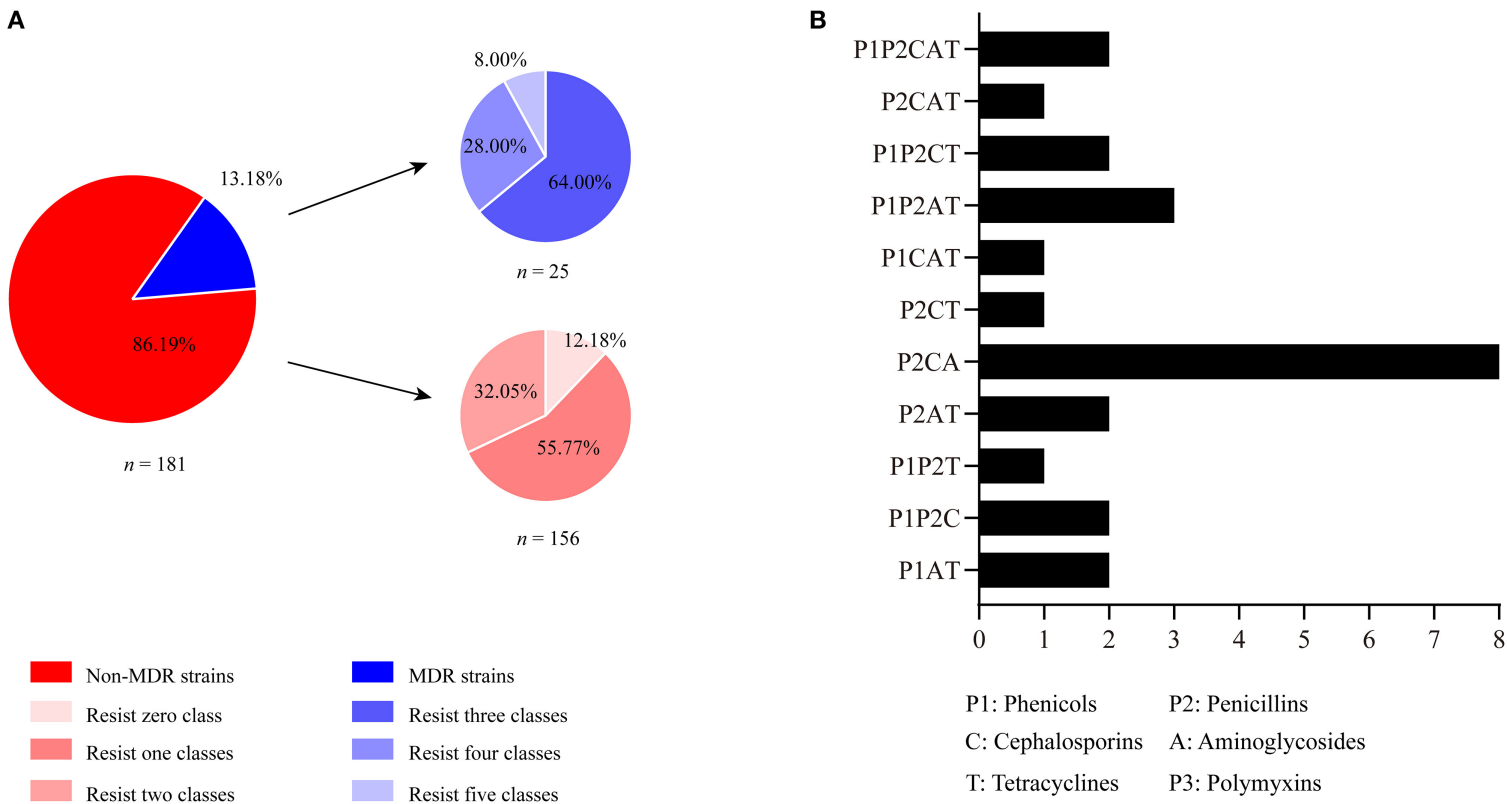
Distribution of multidrug resistant (MDR) strains and non-MDR strains *of B. bronchiseptica* from pigs in China. **(A)** Shows the percentages of MDR and non-MDR strains as well as percent strains resisting 0, 1, 2, 3, 4, and 5 classes of drugs; **(B)** displays the number of isolates resistance to different groups of drug classes. In **(B)**, X axis shows the number of *B. bronchiseptica* strains while Y axis indicates different resistance patterns.

The tested antibiotics in the present study could be divided into eight classes: aminoglycosides (AMK, GEN, TOB), broad-spectrum-cephalosporins (CRO, CTX, CPM), carbapenems (IPM, MRP), fluoroquinolones (ENR, CIP), phenicols (CHL, FLO), penicillins (AMX, AMP), tetracyclines (TET), and polymyxins (PMB). Most of the *B. bronchiseptica* isolates (86.19%, *n* = 156) in this study were resistant to less than three classes of the antibiotics. Among these isolates, 55.77% (*n* = 87) and 32.05% (*n* = 50) of them were resistant to one and two classes of drugs, respectively (Figure 3A). Approximately 13.18% (*n* = 25) of the isolates were resistant to more than three classes of the antibiotics. According to the international expert proposal for interim standard definitions for acquired resistance (32), these 25 *B. bronchiseptica* isolates could be defined as multidrug resistant (MDR) strains. Among these MDR strains, proportions of isolates resistance to three-, four-, and five-classes of drugs were 64.00% (*n* = 20), 28.00% (*n* = 7), and 8.00% (*n* = 2), respectively (Figure 3A). Most MDR-strains possessed a phenotype of co-resistance to aminoglycosides, broad-spectrum-cephalosporins, and penicillins (37.93%, *n* = 11) (Figure 3B).

### Detection of Antimicrobial Resistance Genes

**Figure 4:**
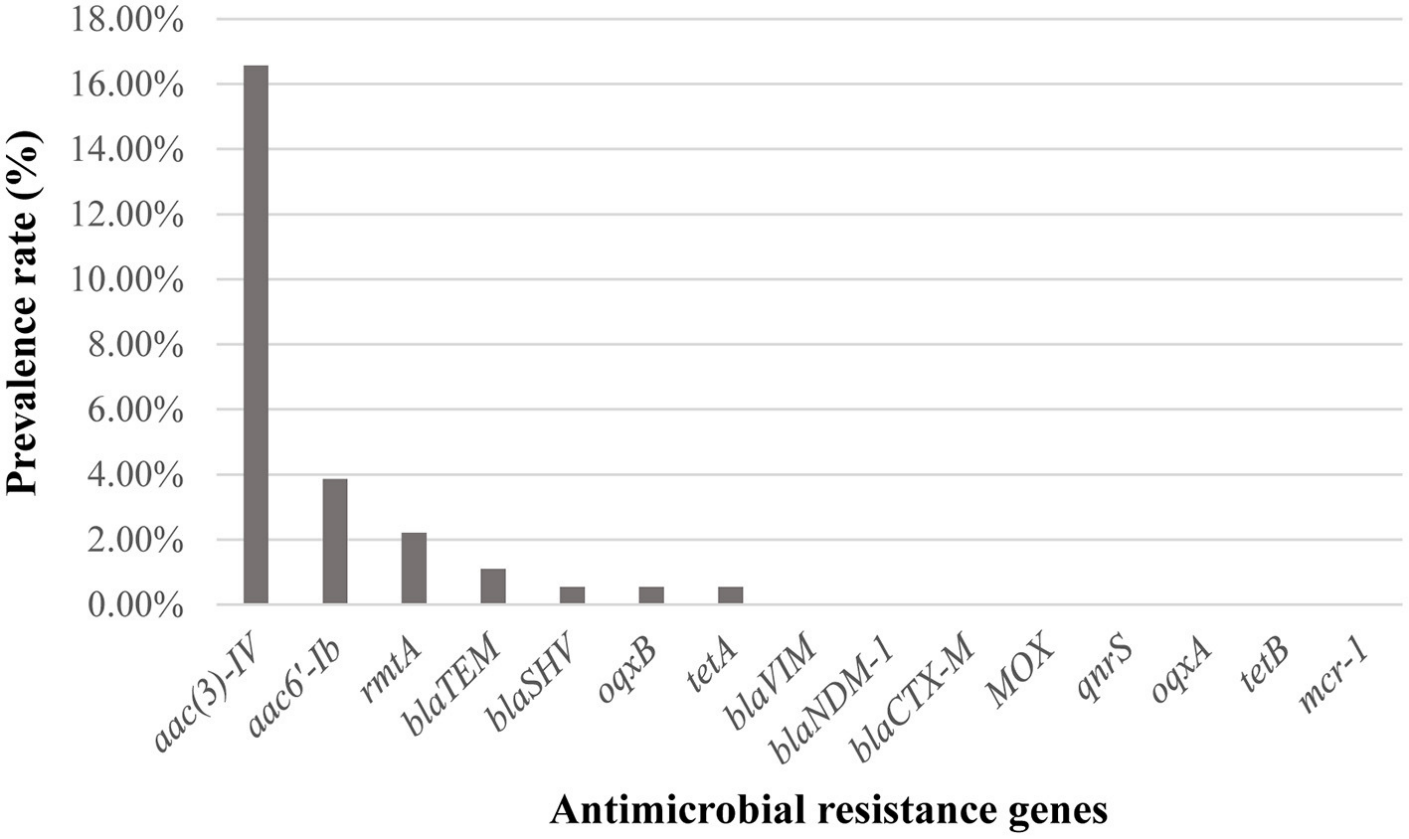
Distribution of antimicrobial resistance genes (ARGs) among *B. bronchiseptica* isolates in this study.

Detection of ARGs showed that 16.57% (*n* = 30) of the *B. bronchiseptica* isolates in this study was positive to *aac(3)-IV*, while 3.87% (*n* = 7), 2.21% (*n* = 4), 1.10% (*n* = 2), 0.55% (*n* = 1), 0.55% (*n* = 1), and 0.55% (*n* = 1) of the isolates were positive to *aac6'-Ib, rmtA, bla*_TEM_, *bla*_SHV_, *oqxB*, and *tetA*, respectively (Figure 4). All isolates were negative to the other ARGs detected (*bla*_VIM_, *bla*_NDM−1_, *bla*_CTX−M_, *MOX, qnrS, oqxA, tetB*, and *mcr-1*).

### Detection of Virulence Factors Encoding Genes

**Figure 5:**
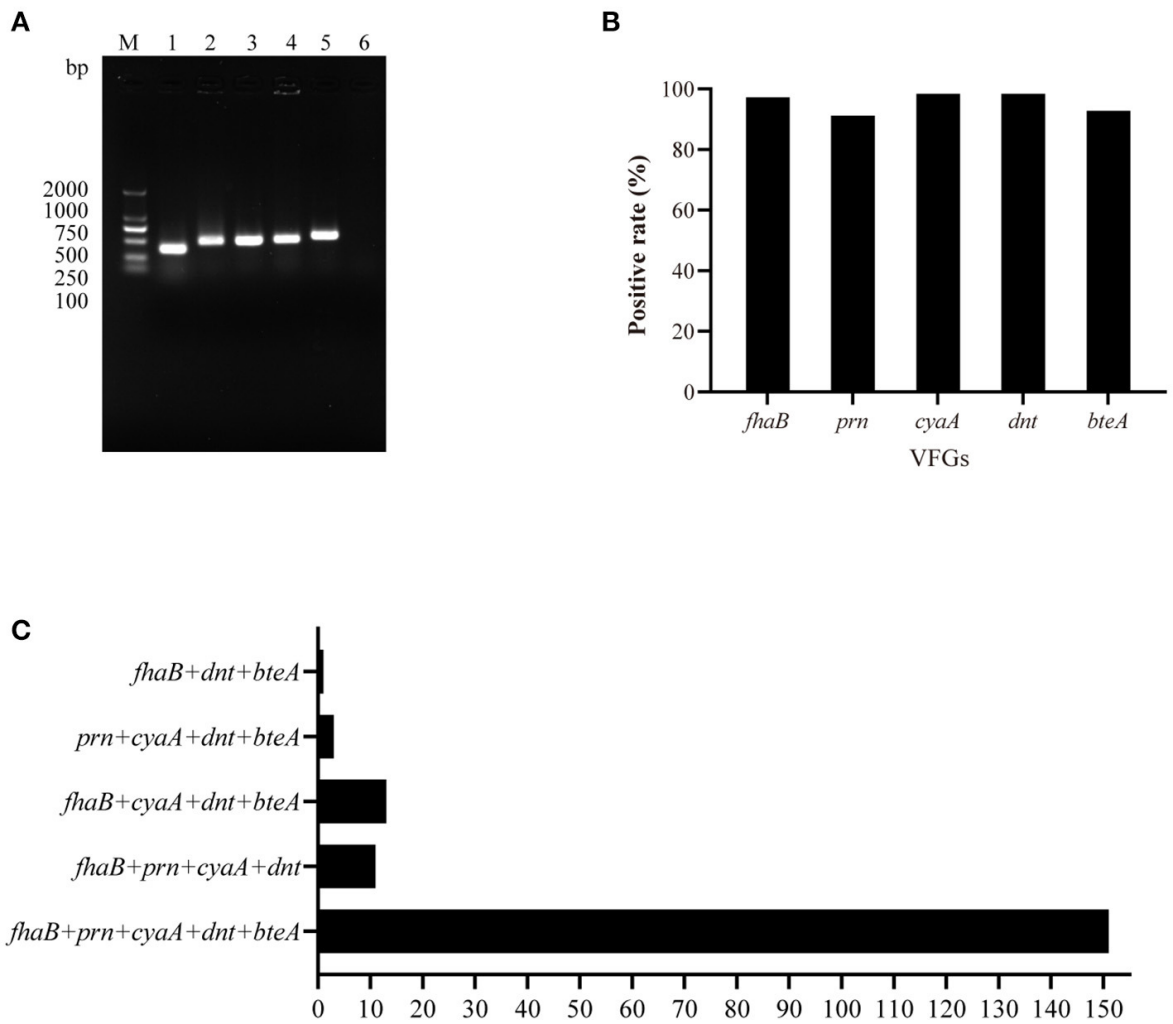
PCR detection of virulence factors encoding genes (VFGs) among *B. bronchiseptica* isolates in this study. **(A)** Shows agarose gel analysis on the PCR products on the five VFGs *cyaA* (band 1, 377 bp), *betA* (band 2, 474 bp), *fhaB* (band 3, 475 bp), *dnt* (band 4, 491 bp), and *prn* (band 5, 555 bp); **(B)** shows the detection rates of the five VFGs while **(C)** shows the number of strains containing different groups of VFGs. In **(C)**, X axis shows the number of *B. bronchiseptica* strains while Y axis indicates different groups of VFGs.

Screening of VFGs revealed that 98.90% (*n* = 179) of the *B. bronchiseptica* isolates in this study was positive to at least one of the five VFGs detected while the remaining 1.10% (*n* = 2) ones were negative to all VFGs. The detection rates of *fhaB, prn, cyaA, dnt*, and *betA* were 97.24% (*n* = 176), 91.16% (*n* = 165), 98.34% (*n* = 178), 98.34% (*n* = 178), and 92.82% (*n* = 168), respectively (Figures 5A,B). Among the VFG-positive isolates, 84.36% (*n* = 151) of the isolates contained *fhaB, prn, cyaA, dnt*, and *betA*, simultaneously (Figure 5C). The remaining isolates harbored “*fhaB*+*prn*+*cyaA*+*dnt*” (6.15%, *n* = 11), “*fhaB*+*cyaA*+*dnt*+*betA*” (7.26%, *n* = 13), “*prn*+*cyaA*+*dnt*+*betA*” (1.68%, *n* = 3), and “*fhaB*+*dnt*+*betA*” (0.56%, *n* = 1), respectively (Figure 5C).

## Discussion

Although *B. bronchiseptica* is a well-known leading cause of pig respiratory disorders and an important causative agent of PRDC, there is not too much report on the epidemiology of *B. bronchiseptica* in pigs round the world, particularly in China, the largest pig rearing and production country. In this study, we described the isolation and characterization of *B. bronchiseptica* in pigs in China from 2018 to 2020. The average isolation rate of this 3-year period was 4.25% (181/4259), which is much lower than that reported in pigs with clinical respiratory disease in China from 2003 to 2008 (4.25 vs. 18.6%, *P* <0.05) (26). The average isolation rates of *B. bronchiseptica* in pigs in different regions from 2018 to 2020 were also much lower than those reported in the same regions from 2003 to 2008 (Hubei: 3.48 vs. 18.0%, *P* <0.05; Henan: 3.42 vs. 19.6%, *P* <0.05; Fujian: 4.14 vs. 18.4%, *P* <0.05; Hunan: 5.96 vs. 19.2%, *P* <0.05; Anhui: 7.32 vs. 18.0%, *P* <0.05; Shandong: 3.98 vs. 20.7%, *P* <0.05) (26). The significant decreasing average isolation rate of *B. bronchiseptica* from 2018 to 2020 compared to that from 2003 to 2008 might be owing to China's continuously efforts to promote transformation and upgrading of pig industry as well as improve the level of disease prevention and control in pig farms. In addition, the outbreak of African Swine Fever in 2018 and its continuous circulation in pigs in China also accelerates the improvement and enhancement of biosecurity on pig farms in recent years (33), which may also be beneficial for the control of *B. bronchiseptica* and the other pathogens.

Administration of antimicrobials is still one of the most effective way to control *B. bronchiseptica* and the other bacteria, but the emergence of drug-resistant bacteria may lead to the failure of using antibiotics in clinic (34–36). Therefore, monitoring the drug resistance profile of clinical microbiology is an important aspect in many epidemiological studies (25, 37, 38). In this study, we characterized the resistance phenotypes of *B. bronchiseptica* from pigs in China from 2018 to 2020. The results revealed that all isolates were susceptible to imipenem (100%), meropenem (100%), and polymyxin B (100%). All of these three types of antibiotics are proposed to be the last-resort antibiotics for the treatment of infections caused by gram-negative bacteria (29), and they are not approved to be used in veterinary medicine in China. In addition, the majority of the isolates were sensitive to ciprofloxacin (99.45%), cefepime (97.79%), enrofloxacin (97.79%), tobramycin (92.27%), gentamicin (86.74%), florfenicol (86.74%), chloramphenicol (86.19%), tetracycline (85.08%), amikacin (83.43%), and amoxicillin (83.43%). These results are in agreement with the results of previous studies in China (25, 39), as well as in other countries such as Germany and Korea (2, 40–42), suggesting these antibiotics might be suitable candidates for treating *B. bronchiseptica* infections when necessary. A high level of resistance was found for ampicillin (83.98%), followed by resistance for cefotaxime (30.39%). These findings are also in agreement with those from the other articles (2, 25, 39), and in particular, *B. bronchiseptica* is documented to be commonly resistant to ampicillin (2). Therefore, these drugs are not recommended to be used in clinic settings. It should be also reminded that several *B. bronchiseptica* isolates from pigs in China displayed a level of multidrug resistance, particularly co-resistance to aminoglycosides, broad-spectrum-cephalosporins, and penicillins. Continues studies should be taken to monitor the prevalence and change-trend of these MDR-isolates in clinic, as some antibiotics belonging to aminoglycosides, broad-spectrum-cephalosporins, and penicillins are commonly used for treating *B. bronchiseptica* infections in veterinary medicine (2, 35).

Virulence factors (VFs) play an important role in the pathogenesis of bacteria (43). For *B. bronchiseptica*, important VFs include filamentous haemagglutinin (FHA), pertactin (PRN), adenylate cyclase-haemolysin toxin, dermonecrotic toxin (DNT), and types III secretion system (44–48), and the expression of these VFs facilitates the invasion of *B. bronchiseptica* in hosts (49). In the present study, we examined five genes encoding these VFs, including *fhaB* which encodes filamentous haemagglutinin; *prn* which encodes pertactin; *cyaA* which encodes adenylate cyclase-haemolysin toxin; *dnt* which encodes DNT; and *bteA* which encodes the T3SS effector A. Surprisingly, over 90% of the pig *B. bronchiseptica* isolates in this study were positive to these five VFGs examined (*fhaB*, 97.24%; *prn*, 91.16%; *cyaA*, 98.34%; *dnt*, 98.34%; *betA*, 92.82%). Importantly, approximately 84.36% of the isolates contained these five kinds of VFGs simultaneously. These results are also in agreement with those reported in *B. bronchiseptica* isolates from rabbits in China (25), suggesting carrying of these VFGs are broad characteristics of *B. bronchiseptica*. Laboratory studies have shown that FHA, and PRN expressed in *E. coli* and *Salmonella* enterica, as well as adenylate cyclase-haemolysin toxin expressed in *B. bronchiseptica* provide protection against fatal infections with *B. bronchiseptica* in mouse models (5, 50, 51).

Despite the findings, this work has several limitations that should be noted. First, all samples used for bacterial isolation were submitted by pig farms from different provinces in China. This way of sample collection may have some influences on the isolation rate. However, the outbreak of African Swine Fever since 2018 and its continuous circulation in pigs in China, and more recently, the worldwide pandemic of the novel coronavirus disease since the late 2019 (COVID-19) made it very difficult for us to collect samples initiatively. Second, the results of antimicrobial susceptibility testing in this study were interpreted by using breakpoints to *Enterobacteriaceae* published in CLSI document M100, and this is because clinic breakpoints specific to *B. bronchiseptica* are limited available (2). Third, a very few published epidemiological studies of swine *B. bronchiseptica* in China are available to date [On March 18, 2021, we searched PubMed with key words “(((*Bordetella bronchiseptica*) AND (Prevalence)) AND (Pigs)) AND (China)” for reports published, with no language restrictions. Our search identified two articles (26, 39) of relevance to this study. All of them were published by our group in 2011], therefore, we only compared the results we obtained from this study to those reported in our previously published two studies in 2011 (26, 39). However, the results from this work could still help understand the current epidemiological and microbiological characteristics of *B. bronchiseptica* in pigs in China.

In summary, we reported the isolation, antimicrobial resistance phenotypes, the detection of ARGs and VFGs of *B. bronchiseptica* from pigs in China from 2018 to 2020 in this study. Our results showed that *B. bronchiseptica* remains an important pathogen associated with pig respiratory disorders in China. While most of the isolates were still susceptible to ciprofloxacin, cefepime, enrofloxacin, tobramycin, gentamicin, florfenicol, chloramphenicol, tetracycline, amikacin, and amoxicillin, MDR-isolates were still determined. These isolates should receive more attentions and further studies are necessary to monitor the prevalence of drug-resistant *B. bronchiseptica*. In addition, our results also revealed that several VFGs, including *fhaB, prn, cyaA, dnt*, and *betA* displayed a high level of detection rate.

## Data Availability Statement

The original contributions presented in the study are included in the article/supplementary material, further inquiries can be directed to the corresponding authors.

## Author Contributions

YZ, ZP, and BW delineated the study conception and design. ZP and BW supervised the study. YZ, HY, LG, MZ, FW, WS, LH, LW, WL, and XT collected the bacterial isolates and performed laboratory tests as well as analyzed the data. ZP and YZ wrote the manuscript and approved the final version for publication. ZP, BW, and WL participated in the manuscript discussion and revision. All authors have read and approved the final version of the manuscript.

## Conflict of Interest

LG and XT were employed by the company Wuhan Keqian Biology Co., Ltd. The remaining authors declare that the research was conducted in the absence of any commercial or financial relationships that could be construed as a potential conflict of interest.
